# Shared Decision-Making Behavior in Surgery Among Early Breast Cancer Patients and Associated Factors Using COM-B Model: A Latent Profile Analysis

**DOI:** 10.1155/jonm/2347796

**Published:** 2025-07-16

**Authors:** Wei Zhang, Ye Weng, Xiaoyang Zhang, Haiyan Shen, Xiaochun Li, Yue Liu, Wei Liu, Han Xiao, Haihong Jing, Chao Xu, Han Tang

**Affiliations:** ^1^School of Nursing, Wenzhou Medical University, Wenzhou, Zhejiang, China; ^2^Department of Oncology, Renmin Hospital of Wuhan University, Wuhan, Hubei, China; ^3^Department of Orthopedics, Sichuan Academy of Medical Sciences and Sichuan Provincial People's Hospital, Chengdu, Sichuan, China; ^4^Medical Department, The First Affiliated Hospital of Air Force Medical University, Xi'an, Shaanxi, China; ^5^Department of Health Statistics, School of Public Health, Air Force Medical University, Xi'an, Shaanxi, China; ^6^Department of Breast Surgery 2 (Area 1), The First Affiliated Hospital of Zhengzhou University, Zhengzhou, Henan, China; ^7^Department of Knee Joint Surgery, Honghui Hospital of Xi' an Jiaotong University, Xi'an, Shaanxi, China

**Keywords:** breast cancer, factor, latent profile analysis, shared decision making, surgery

## Abstract

**Background:** Breast cancer patients often face the choice between breast-conserving surgery and mastectomy. From a shared decision-making perspective, it is crucial for patients to actively engage in the decision-making process, taking into account their own preferences and values. This approach can enhance treatment satisfaction and support the principles of precision nursing.

**Objective:** To evaluate the level and classification of shared decision-making behavior in surgery, along with the influence of participation competence, perceived social support, and self-care self-efficacy, guided by the “Capacity, Opportunity, Motivation-Behavior” model.

**Method:** A multicenter cross-sectional study was conducted in three hospitals in China from January 2021 to March 2022. The survey tools included self-designed demographic and clinical instrument, the Participation in Treatment Decision-Making Scale for cancer patients (PTDMS), the Participation Competence Scale (PCS), the Perceived Social Support Scale (PSSS), and the Strategies Used by People to Promote Health (SUPPH). Latent profile analysis was employed to assess the shared decision-making behavior in surgery. Multivariate logistic regression was applied to identify factors associated with the identified subtypes.

**Result:** A total of 840 participants were ultimately analyzed. The best-fitting model identified three classes: active participation group (55.7%), moderate participation group (21.7%), and low participation group (22.6%). Logistic regression indicated that age, number of children, educational level, family income, employment status, cancer stage, type of surgery, participation competence, perceived social support, and self-care self-efficacy were main associated factors (all *p* < 0.05).

**Conclusion:** The performance of shared decision-making behaviors in surgery needs improvement. This study may help nurses identify targeted intervention populations who are older, have more than three children, have a higher education level, have a lower family income, are employed, are at an advanced cancer stage, and are opting for mastectomy. It also emphasizes the importance of participation competence, social support, and self-care self-efficacy when designing intervention content.

## 1. Introduction

The incidence of breast cancer (BC) is significant, with 2.3 million new cases reported among women worldwide in 2022. Half of the 185 countries and territories surveyed experienced an annual increase of 1%–5% in cases [1]. In addition, the global incidence of BC is trending younger [1], especially in China, where the incidence of BC in women under 35 years of age is increasing [2] and a large proportion of these cases are classified as early stage [3]. The diversity and effectiveness of treatments have also improved the overall survival of early BC, with surgery being one of the most common and important treatments. When early BC patients choose whether breast-conserving surgery or mastectomy, they need to consider not only prognosis but also their personal preferences, such as concerns about body image and cost, and on the other hand, they have a great psychological burden about the change in body image after total mastectomy [4]. As a result, conflicts and challenges in surgical decision making are common among BC patients.

In this process, nurses have the closest contact with patients and play a crucial role. Through frequent communication in their daily nursing work, they can keenly perceive various forms of confusion and ambivalence in patients' surgical decision-making processes [5]. Nurses also provide detailed information about surgical procedures, helping patients understand the pros and cons of different options. Additionally, they utilize their professional communication skills to offer emotional support and encourage patients to express their true feelings, thereby enhancing their participation in the decision-making process. Conducting study from the nursing perspective can yield insights that may be overlooked by other medical specialties and provide a more comprehensive strategy for addressing surgical decision-making conflicts and challenges in BC patients. Furthermore, nursing staff serve as a vital bridge within the healthcare team, offering accurate feedback on patients' requests to other team members, such as physicians. This collaboration facilitates the provision of more appropriate treatment options, ultimately enhancing patient satisfaction with the surgical decision-making process and improving the overall treatment experience and prognostic quality of life.

With the transformation of the patient-centered medical model and the promotion of precision nursing, the benefits of treatment shared decision making (SDM) have been gradually verified in recent years. These benefits include improvements in sexual relationships between spouses, medical satisfaction, social participation, and overall quality of life [6]. Furthermore, the format of SDM varies across different regions and cultures. Influenced by Confucian culture, Chinese female patients typically receive significant support from their families during illness, often resulting in family members holding more authority than the patients themselves [7]. This dynamic can impact patients' genuine thoughts and feelings, leading to a higher occurrence of surrogate decision making in China [8]. However, influenced by liberal ideologies, the individual consciousness of patients has been awakening in recent years. Participating in treatment SDM can be considered a form of health behavior change [9]. Several studies [10, 11] have shown that the willingness to participate in treatment SDM among BC patients is increasing, but evidence regarding actual SDM behavior remains insufficient. For instance, a study [12] assessing early BC females in Massachusetts and Minnesota revealed that 78.2% of participants reported having a say in their surgical decisions and expressed satisfaction with the outcomes. Conversely, another study [13] found that fewer than 35% of cancer patients in Minnesota actively participated in the decision-making process. Therefore, it is essential to explore SDM behavior in surgery among Chinese early BC patients, considering their specific cultural backgrounds and theoretical frameworks.

Given that treatment SDM participation can be regarded as a type of health behavior change, the well-regarded “Capacity, Opportunity, Motivation-Behavior model” (COM-B) could guide the selection of potential influencing factors [14]. This classic and comprehensive theory posits that to achieve a specific health behavior change, individuals require both internal (capacity and motivation) and external factors (opportunity), with these three elements working together to influence health behavior. In this study, we focused on SDM behavior in surgery. Capacity (C) refers to participation competence, which encompasses an individual's ability to obtain information, communicate effectively, access resources for making independent decisions, and manage emotions during the decision-making process. Opportunity (O) refers to perceived social support, which reflects individuals' subjective evaluation and emotional experiences during social interactions. Motivation (M) pertains to self-care self-efficacy, meaning the individual's belief and confidence in effectively managing themselves to achieve desired outcomes in specific situations or tasks.

Regarding demographic and clinical factors, research on treatment SDM behavior among BC patients is limited. For instance, females with higher educational levels tend to participate more actively in their cancer treatment decision making [10]. As for factors such as marital status and the number of children, some studies [15, 16] indicate that marital status can influence decision making about salpingo-oophorectomy, and the number of children also warrants further emphasis, although evidence is scarce. These factors are important and should be evaluated further. The effects of family income on participation in different treatment SDM behaviors among BC patients have produced controversial results [17, 18]. Furthermore, the impact of cancer stage has shown statistically significant effects in some studies [11] but is absent in others. Regarding research methods, while some study [19] has measured treatment SDM behavior using scales, they did not classify the levels of SDM behavior in surgery, resulting in a vague understanding of its distribution within the BC population and low specificity in associated factors.

Promoting BC patients' SDM behavior in surgery is not only a key goal of Healthy China 2030 but also a positive response to the concept of precision nursing. Therefore, from the perspective of health behavior change, this study aimed to evaluate the level and subtypes of BC patients' SDM behavior in surgery within the context of Chinese Confucian culture. Additionally, it sought to explore the influence of various comprehensive factors guided by the COM-B model, providing a scientific basis for intervention design. The research hypothesis posited that the level of SDM behavior in surgery could be divided into different subtypes. Furthermore, several unclear or controversial demographic and clinical characteristics, along with females' participation competence, self-care self-efficacy, and perceived social support, which are selected from the COM-B model, would significantly influence the SDM behavior in surgery among BC patients.

## 2. Materials and Methods

### 2.1. Study Population

Using a convenience sampling method, patients with BC from three Grade 3A hospitals in Xi'an and Zhengzhou from January 2021 to March 2022 were selected. The inclusion criteria were as follows: (1) aged 18 years or older; (2) diagnosed with BC through pathological examination; (3) at an early cancer stage (0-II), meeting the criteria for both breast-conserving surgery and mastectomy; (4) either undergoing or having completed surgery decision making; (5) possessing clear consciousness and the ability to read, write, and communicate verbally in daily life; and (6) understanding the study's purpose and consenting to participate. The exclusion criteria included (1) selection of only one type of surgery due to personal or other reasons; (2) a history of mental illness, severe cognitive impairment, significant visual, auditory, or language impairments; (3) severe heart or liver function impairments or other serious comorbidities; (4) current participation in similar studies; and (5) lack of consent from family members. The three centers involved in this study are located in provincial capital cities in Central and Western China, which have a relatively large regional influence and an abundant pool of patients. Based on sample size calculations using latent profile analysis (LPA) and unordered multicategorical logistic regression, at least 690 patients were required (the sample size was set at 5–10 times the number of research variables, considering our study included 3 centers and accounting for a 20% loss to follow-up). Ultimately, 900 questionnaires were distributed. This study was approved by the Ethics Committee of the Affiliated Hospital of Zhengzhou University (No: 2020-KY-220).

### 2.2. Study Procedure

A total of 11 research members in this study consisted of two study managers, four research assistants (mainly for questionnaire distribution and collection), two quality control staffs, and three data entry and statistical analysts. All research members were required to undergo unified training before conducting this research. The training covered the purpose of the study, the structure of the questionnaire, filling norms, and key points for effective communication with patients. Following this, a face-to-face survey was conducted. After confirming participants' eligibility based on the inclusion criteria, the research members approached the patients and explained the purpose of the study. Once the patients agreed to participate, they were asked to sign the informed consent form, which was collected by the research members. The patients then filled out the questionnaires independently and the time taken to complete the questionnaire was controlled to 10–15 min. During this process, if the patients had any questions about the items, the researchers provided explanations. After the patients completed the questionnaires, they returned them on the spot, and the members checked for any missing items, blanks, unclear responses, and overly uniform or indecipherable content. If any issues were found, the patients were asked to provide the necessary information promptly. For certain clinical information that patients were unsure about, the research members verified it using the hospital's medical record system. The verified data were then entered into the system using two-person double entry for subsequent analysis.

### 2.3. Assessment Tools

#### 2.3.1. Demographic and Clinical Instrument

The self-designed demographic and clinical instrument was developed by consulting relevant literature and experts. It included demographic variables such as age, educational level, marital status, number of children, religion, type of health insurance, household per capita monthly income, and employment status. Clinical variables included cancer stage, whether the disease was unilateral or bilateral, and the type of surgery performed.

#### 2.3.2. Participation in Treatment Decision-Making Scale of Cancer Patients (PTDMS)

It was originally developed in 2003 [20] and introduced to China in 2004, to measure the actual treatment SDM participation behavior of cancer patients. This tool had single dimension and contains 12 items. All items adopted the Likert 3-point scoring, with scores ranging from “1” to “3” points assigned, respectively, from “participate a lot” to “not participate.” An average score of items ≥ 1.5 points indicated poor participation behavior. The Cronbach'*α* coefficient in this study was 0.872.

#### 2.3.3. Participation Competence Scale in Decision Making (PCS)

It was developed from a Chinese scholar in 2012 and was used to evaluate the subjective and objective abilities required by cancer patients during the treatment process. It included 4 dimensions and 31 items, namely, “information acquisition” (6 items), “independent decision making” (6 items), and “communication” (7 items) and “emotional management” (12 items). All items adopted the Likert 5-point scoring, with scores ranging from “1” to “5” points assigned, respectively, from “strongly disagree” to “strongly agree.” The total score ranged from 31 to 155 points, and the higher the score, the higher the participation competence of cancer patients during the treatment SDM process. The Cronbach'*α* coefficient in this study was 0.907.

#### 2.3.4. Perceived Social Support Scale (PSSS)

It was developed in 1987 [21] to evaluate the level of social support perceived by individuals from families, friends, leaders, relatives, colleagues, etc. This scale included 3 dimensions with a total of 12 items, namely, “family support” (4 items), “friend support” (4 items), and “other support” (4 items). All items adopted the Likert 7-point scoring, with scores ranging from “1” to “7” points assigned, respectively, from “strongly disagree” to “strongly agree.” The total score ranged from 12 to 84 points, and the higher the score, the higher the level of subjective social support perceived by the subjects. The Cronbach'*α* coefficient in this study was 0.951.

#### 2.3.5. Strategies Used by People to Promote Health (SUPPH)

It was designed in 1996 [22], including 3 dimensions and 28 items, namely, “self-stress reduction” (10 items), “self-decision making” (3 items), and “positive attitude” (15 items). All items adopted the Likert 5-point scoring method, with scores ranging from “1” to “5” points assigned, respectively, from “strongly disagree” to “strongly agree.” The total score ranged from 28 to 140 points, and the higher the score, the higher the self-care self-efficacy of cancer patients. The Cronbach'*α* coefficient in this study was 0.938.

### 2.4. Statistical Methods

Data were imported into EpiData 3.0, and statistical analysis was performed using SPSS 26.0 and Mplus 8.3. For measurement data conforming to the normal distribution, the mean ± standard deviation was used. For counting data, the number of cases and percentage (%) were used for representation. The chi-square test or Fisher's exact probability method was used for the comparison between unordered categorical variables, and the Kruskal–Wallis *H* test was used for the comparison between unordered categorical variables. The significance level *α* = 0.05. LPA has been employed to explore the underlying group structure within the data and classify individuals into multiple groups, providing a foundation for selecting targeted populations that fall into poor performance classifications. This can help determine key populations and the necessary content for precise decision-making optimization interventions. LPA was conducted with the 12 items of the PTDMS as the explicit indicators to extract the characteristics of BC patients' surgery SDM behavior. The following indicators were used to evaluate the model fitting effect: (1) the Akaike information criterion (AIC), Bayesian information criterion (BIC), and adjusted Bayesian information criterion (aBIC) were used as fitting indicators. The smaller the values, the better the model fitting effect [23]. (2) Entropy was used as the classification indicator. Entropy ranges from 0 to 1, and the larger the value, the more accurate the classification. Generally, a model with an entropy value reaching above 0.8 is acceptable. (3) The Lo–Mendell–Rubin likelihood ratio test (LMR) and the bootstrapped likelihood ratio test (BLRT) were used as likelihood ratio test indicators. If *p* < 0.05, it indicates that the *n*-category model is better than the *n*-1-category model. Taking the score of surgery SDM behavior among BC patients as the dependent variable to explore its associated factors. After conducting the parallel lines analysis, ordered multinomial logistic regression would be used if *p* > 0.05; otherwise, unordered multinomial logistic regression would be selected. A difference was considered statistically significant when *p* < 0.05.

## 3. Results

### 3.1. Demographic and Clinical Characteristics of BC Patients

A total of 900 questionnaires were distributed, and 840 valid responses were received, resulting in an effective recovery rate of 93.3%. Reasons for drop-out included unavailability due to time constraints (*n* = 25), temporary transfer to another hospital due to worsened conditions (*n* = 17), physical discomfort (*n* = 10), and family refusal (*n* = 8). All participants were female and among them, 29.0% (*n* = 244) patients were 31–40 years, 80.1% (*n* = 673) were married, 38.6% (*n* = 324) had two children, and 82.5% (*n* = 693) had no religion. Furthermore, 50.7% (*n* = 426) were senior high school and junior college, 83.5% (*n* = 701) had new rural cooperative medical system, 8.1% (*n* = 68) reported the household per capita monthly income of less than 1000 yuan, and 32.6% (*n* = 274) were not in employment. In terms of clinical characteristics, 48.0% (*n* = 403) were in II stage of BC, 73.6% (*n* = 618) were unilateral BC, and 66.3% (*n* = 557) received mastectomy.

### 3.2. Latent Profile Categories and Characteristics of SDM Behavior in Surgery

Table 1 shows the data of 12 items from the PTDMS, which were used for LPA analysis to establish latent profile models ranging from 1 to 5 categories. As the number of categories increased from 1 to 5, the values of AIC, BIC, and aBIC of the models all decreased. Among them, the 3-category model was superior to the 2-category model. Moreover, when testing the 4-category model, the LMR value was not significant (*p* > 0.05), indicating that the 4-category model was not better than the 3-category model. Judging from the entropy index, the entropy values in different categories were all greater than 0.9, and the entropy value of the 3-category model reached 0.98. Considering both the LMR value and the entropy index, the 3-category model was determined.

**Figure 1:**
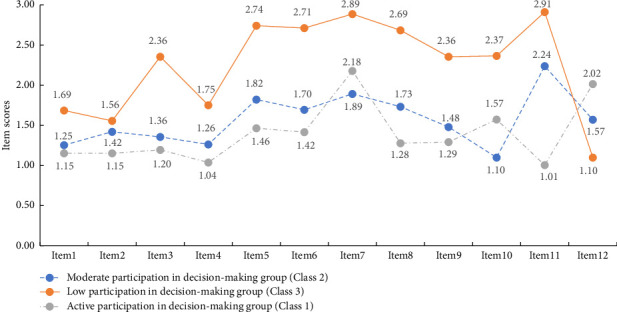
Three-category latent profile analysis of participation in surgery shared decision-making behavior.

Figure 1 shows that the overall characteristics of BC patients' surgery SDM behavior could be divided into three categories. There were 182 patients in the first category (the middle line in Figure 1), accounting for 21.7% of the total. Most of points in the middle line (Figure 1) of the item scores were intermediate except item 7 and item 10, indicating that the level of SDM behavior in surgery of participants was moderate. Therefore, this category was named the “moderate participation group” (Class 2). There were 469 patients in the second category (the top line in Figure 1), accounting for 55.7% of the total. Except for item 12, all the item scores of participants were the highest, suggesting that the level of surgery SDM behavior of these participants was high. Hence, this category was named the “active participation group” (Class 1). There are 189 patients in the third category (the bottom line in Figure 1), accounting for 22.6% of the total. The item scores of participants were all at the lowest and intermediate levels, and most of the item scores were the lowest, indicating that the level of SDM behavior in surgery of participants was the lowest. Therefore, this category was named the “low participation group” (Class 3).

Table 2 shows the comparison of each item scores, and the total scores among three subtypes were all statistically significant (*p* < 0.001). The item scores of the “low participation group” were basically lower than “moderate participation group” and “active participation group.” Except for item 12, the item scores of the “active participation group” were lower than those of “moderate participation group.”

### 3.3. Univariate Factors of Surgery SDM Behavior Among BC Patients

Table 3 shows that there were statistically significant differences (*p* < 0.01) among the three subtypes of BC patients in terms of age, marital status, number of children, living environment, educational level, household per capita monthly income, employment status cancer stage, unilateral or bilateral breast diseases, type of surgery, perceived social support, participation competence, and self-care self-efficacy.

### 3.4. Multivariate Factors of SDM Behavior in Surgery Among BC Patients

Taking the three subtypes of SDM behavior in surgery among BC patients as the dependent variable, the indicators with statistically significant differences in the univariate analysis (*p* < 0.05) were used as independent variables for multivariate analysis.  Table 4 shows the assignment of independent variables.

Table 5 shows that when comparing Class 2 with Class 1, age (OR = 3.155, 95% CI: 1.192∼8.348), household per capita monthly income (OR = 9.644, 95% CI: 3.772∼24.661; OR = 11.437, 95% CI: 5.012∼26.100), and perceived social support (OR = 1.068, 95% CI: 1.036∼1.100) were risk factors in Class 2. Having no child (OR = 0.315, 95% CI: 0.111∼0.894), no work (OR = 0.205, 95% CI: 0.097∼0.432), breast-conserving surgery (OR = 0.344, 95% CI: 0.172∼0.688), participation competence (OR = 0.921, 95% CI: 0.886∼0.957), and self-care self-efficacy (OR = 0.893, 95% CI: 0.857∼0.931) were positive factors for Class 2. When comparing Class 3 with the Class 1, 31–40 years (OR = 4.834, 95% CI: 1.241∼18.381), educational level (OR = 56.992, 95% CI: 12.480∼260.571; OR = 46.281, 95% CI: 11.982∼178.754), household per capita monthly income (OR = 10.867, 95% CI: 2.144∼55.080; OR = 3.923, 95% CI: 1.279∼12.028), and at 0 cancer stage of BC (OR = 15.787, 95% CI: 3.197∼75.900) were risk factors in Class 3. Age of 18–30 years (OR = 0.809, 95% CI: 0.764∼0.856), participation competence (OR = 0.809, 95% CI: 0.764∼0.856), and self-care self-efficacy (OR = 0.846, 95% CI: 0.894∼0.901) were positive factors for Class 3. When comparing Class 3 with the Class 2, 41–50 years (OR = 7.669, 95% CI: 1.929∼30.438), educational level (OR = 105.470, 95% CI: 25.255∼440.463; OR = 45.429, 95% CI: 13.028∼156.250), no work (OR = 4.557, 95% CI: 1.621∼12.921), at 0 cancer stage of BC (OR = 10.720, 95% CI: 2.678∼42.907), and breast-conserving surgery (OR = 4.876, 95% CI: 1.824∼13.033) were risk factors in Class 2. 1000–3000 yuan per month (OR = 0.154, 95% CI: 0.055∼0.432), participation competence (OR = 0.878, 95% CI: 0.837∼0.922), perceived social support (OR = 0.959, 95% CI: 0.933∼0.987), and self-care self-efficacy (OR = 0.948, 95% CI: 0.908∼0.990) were positive factors for Class 2.

## 4. Discussion

To our knowledge, this may be the first study to assess the levels and subtypes of SDM behavior in surgery among Chinese BC patients using LPA and guided by the COM-B model. This research confirms the concept of precise nursing and provides a basis for targeted decision-making support interventions.

The results of this study indicated that the subtype of SDM behavior in surgery included three classes. Participants in Class 1 accounted for 55.7%, which was higher than findings from another study [10] in Germany involving cancer patients, and the percentage of Class 2 participants versus Class 3 participants was similar to the results of this study. Additionally, the level of surgery SDM behavior in this study was unsatisfactory, with an average item score exceeding 1.5 points. This is worse than results from a UK study [24] focusing on surgical decision making among elderly BC patients. A probable reason for this difference is that patients in developed countries tend to have a higher individual awareness of self-control, influenced by their social environment. In contrast, this awareness has only recently begun to awaken among Chinese females, where Confucian culture and family values continue to play significant roles. Furthermore, the sample size in the latter study was only 35 participants, which may have led to result bias. Additionally, another Chinese study [25] on BC patients facing chemotherapy drug decision making reported lower levels of participation of SDM behavior than our study. This discrepancy may be due to the more complex and professional nature of chemotherapy drug choices, which places higher demands on patients' objective abilities to participate in decision making and necessitates greater assistance from healthcare professionals. This underscores the need for more studies in the future to assess the level of treatment SDM behavior, utilizing larger sample sizes, diverse cultural backgrounds, and various decision-making contexts.

There were three crosses spanning three classes in Figure 1. For item 7 (doctors and nurses listen to my opinions related to my treatment) and item 10 (I participate in the discussion of the treatment decision-making process), Class 1 exhibited significantly lower participation behavior than Class 2. This finding contrasts with existing research studies [26], which indicate that trust and effective communication between health professionals and patients, along with patients' high initiative for participation, are key factors in improving SDM behavior among BC patients. However, the previous studies did not employ LPA and did not specifically classify the assessed BC patients. Therefore, it remains unclear whether all groups demonstrated good communication and a high initiative to enhance the level of SDM behavior in surgery. In this study, the tool used to evaluate the level of surgery SDM behavior was self-rated. Importantly, in China, although preoperative informed consent is commonly practiced, actual participation in treatment decision making is relatively low, with most decisions still led by health professionals. This disparity may account for the differences observed in the scores for these two items. Additionally, for item 12 (the doctor decides on my treatment plan), Class 1 had the highest score, indicating low participation, while Class 3 had the lowest score, indicating high participation. Although there were crosses in Figure 1, they align with the practical meaning of Chinese culture, which is consistent with the findings of another [27]. The reason for this is that the strong sense of autonomy among Chinese patients tends to diminish during treatment, leading to greater dependence on health professionals. Consequently, the implementation of SDM in real clinical settings is not particularly significant. Furthermore, female patients may be relatively more emotionally sensitive and delicate, often seeking support from their families and health professionals when making surgery decisions [26]. This suggests a need for more subtype studies to be conducted, and it is essential to verify whether the changing trends are consistent across different classes and items.

In addition, among the factors identified based on the COM-B model, the results indicated that participation competence, perceived social support, and self-care self-efficacy were significant protective factors for SDM behavior in surgery across two different subtypes, with the exception of self-care self-efficacy between Class 3 and Class 1 (*p* > 0.05). Self-care self-efficacy also functioned as a risk factor between Class 2 and Class 1. Patients with high self-care self-efficacy tend to consider not only their medical conditions but also various factors such as economic and social implications, leading to increased hesitation and complexity when making decisions. Higher self-care self-efficacy is associated with greater self-confidence, which may encourage patients to question and evaluate the decisions made by health professionals, potentially impacting their engagement in treatment SDM behavior [28].

In terms of demographic and clinical characteristics, younger, fewer-children, and better-educated BC patients showed higher levels of SDM behaviors in relation to surgery. This is consistent with the effects of age, number of children, and education on actual participation behavior of BC patients [29–31], as younger, fewer-children, and better-educated patients are more adept at obtaining information about their disease through a variety of channels (including traditional media and the Internet), and they have a deeper knowledge of their cancer and an increased ability to comprehend this information, all of which enhance the levels of surgical SDM behavior. Furthermore, we discovered that a lower cancer stage was associated with a lower level of surgery SDM behavior, which contrasts with findings in patients with advanced lung cancer [32]. This discrepancy may be attributed to the more aggressive nature of lung cancer; patients with a higher cancer stage often face a more straightforward treatment regimen due to limited options. Interestingly, the relationship between family income and the level of surgery SDM behavior among BC patients was inconsistent. Most patients with lower income levels exhibited lower participation. However, in the comparison between Class 3 and Class 2, those with an income between 1000–3000 yuan had a higher level of surgery SDM behavior than those earning more than 5000 yuan. This finding differs from another study [33] involving newly diagnosed early BC patients and may be due to our subjects being exclusively from Grade 3A hospitals, leaving unclear the circumstances of patients from community and primary hospitals, which could result in varying impacts of family income on surgery SDM behavior. Moreover, this study found that patients who were unemployed and received breast-conserving surgery were protective factors when comparing Class 2 and Class 1. Conversely, they were considered risk factors when comparing Class 3 and Class 2. This contrasts with results from another study [34] on adjuvant whole breast irradiation among Danish BC patients. A possible explanation is that Chinese patients may be more influenced by Confucian culture, which emphasizes the importance of collectivism. Consequently, women may tend to defer decision-making authority to their families or healthcare professionals, placing themselves in a more passive role.

## 5. Limitations

Firstly, the sample was drawn from only three Chinese Grade 3A hospitals. There may be variations in culture, economic levels, and medical quality in other regions and hospital types, suggesting that the representativeness of the sample should be strengthened in future studies. Secondly, our study was a cross-sectional design, which cannot establish causal relationships. Thirdly, the method of LPA relies on assumptions such as the independence of latent classes and the compliance of data with a specific distribution. However, in clinical settings, female's decision making regarding surgery may be influenced by the interaction of multiple factors, and there can be cross-influences between classes. This may blur the boundaries of the latent classes and may not align with the model's assumptions; thus, the interpretation of results should closely consider practical implications. Lastly, our selection of influencing factors was based on the COM-B theoretical model. Future research is needed to explore underlying factors through additional theoretical models and to investigate a more comprehensive range of influencing factors by incorporating qualitative interviews, mixed-methods research, mediating effects, and other research methodologies.

## 6. Conclusion

Under the theoretical guidance of the COM-B model, we evaluated the level and classification of SDM behavior in surgery among women with early BC in the context of Confucian culture and further explored its associated factors. The level of SDM behavior in surgery in this study was divided into three groups (low, moderate, and active), with the proportion of each group ranging from 21.7% to 55.7%, indicating a need for improvement. Risk factors identified included older age, a larger number of children, lower education levels, and advanced cancer stage. Conversely, protective factors included having no children, higher family income, greater participation competence, perceived social support, and self-care self-efficacy. This study may provide a reference for identifying targeted intervention populations, specifically those who are older, have more than three children, have a lower family income, and present with advanced cancer stages. Additionally, when designing the content of precise interventions, nursing staff should emphasize the elements of participation competence, perceived social support, and self-care self-efficacy.

## Figures and Tables

**Table 1:** Fitting indices of the latent profile model for shared decision-making behavior in surgery.

Model	*k*	AIC	BIC	aBIC	*p*		Entropy	Class probability
					LMR	BLRT		
1	24	21,615.538	21,729.14	21,652.924				
2	37	17,152.49	17,327.626	17,210.126	0.969	< 0.001	< 0.001	0.40180/0.59820
3	50	16,559.92	16,796.591	16,637.807	0.980	0.020	< 0.001	0.21713/0.55685/0.22602
4	63	15,967.403	16,265.607	6065.54	0.992	0.0816	< 0.001	0.13420/0.11692/0.22500/0.52389
5	76	15,035.028	15,394.766	15,153.415	0.991	0.5849	< 0.001	0.28041/0.10946/0.11245/0.206006/0.291696

*Note:* aBIC, sample size–adjusted Bayesian information criterion; LMR, Lo–Mendell–Rubin adjusted likelihood ratio test.

Abbreviations: AIC, Akaike information criterion; BIC, Bayesian information criterion; BLRT, bootstrap likelihood ratio test.

^∗^ *p* < 0.05: The *n*-category model is significantly superior to the *n*-1-category model.

**Table 2:** Score differences of relevant items and total scores of the three variables for the evaluation of shared decision-making behavior in surgery ($\overset{¯}{x}\pm s$).

Item	Active participation group (Class 1)	Moderate participation group (Class 2)	Low participation group (Class 3)	*F*	*p*
1. Encourage me to participate in the selection of the treatment plan.	1.15 ± 0.44	1.25 ± 0.44	1.69 ± 0.67	131.192	< 0.001
2. Propose various treatment options to me.	1.15 ± 0.36	1.42 ± 0.53	1.56 ± 0.60	70.072	< 0.001
3. Determine the treatment plan according to my suggestions.	1.20 ± 0.40	1.36 ± 0.55	2.36 ± 0.54	456.133	< 0.001
4. I choose among various treatment plans.	1.04 ± 0.19	1.26 ± 0.44	1.75 ± 0.70	204.866	< 0.001
5. My wishes can be considered in the process of treatment decision-making process.	1.46 ± 0.62	1.82 ± 0.73	2.74 ± 0.48	410.693	< 0.001
6. I decide on the treatment plan myself.	1.42 ± 0.56	1.70 ± 0.59	2.71 ± 0.45	472.269	< 0.001
7. Doctors and nurses listen to my opinions related to my treatment.	2.18 ± 0.49	1.89 ± 0.61	2.89 ± 0.31	442.122	< 0.001
8. I jointly decide my treatment plan with the doctor.	1.28 ± 0.52	1.73 ± 0.66	2.69 ± 0.47	468.868	< 0.001
9. Doctors and nurses seek my opinion before starting the treatment.	1.29 ± 0.52	1.48 ± 0.50	2.36 ± 0.48	425.235	< 0.001
10. I participate in the discussion of the treatment decision-making process.	1.57 ± 0.60	1.10 ± 0.28	2.37 ± 0.74	353.039	< 0.001
11. I express my own views related to own treatment plan.	1.01 ± 0.01	2.24 ± 0.43	2.91 ± 0.29	676.776	< 0.001
12. The doctor decides on my treatment plan.	2.02 ± 0.69	1.57 ± 0.66	1.10 ± 0.30	308.352	< 0.001
Total average score	1.44 ± 0.18	1.63 ± 0.21	2.43 ± 0.19	629.976	< 0.001

**Table 3:** Univariate analysis of latent profile analysis of breast cancer patients' shared decision-making behavior in surgery (*n* = 840).

Variable	Total (*n* = 840, %)	Class 1 (*n* = 469, %)	Class 2 (*n* = 182, %)	Class 3 (*n* = 189, %)	*H*/*χ*^2^ value	*p*
Age (years)					*H* = 29.400	< 0.001^∗^
18–30	281 (33.5)	131 (27.9)	68 (37.4)	82 (43.4)		
31–40	244 (29.0)	143 (30.5)	64 (35.2)	37 (19.6)		
41–50	208 (24.8)	127 (27.1)	29 (15.9)	52 (27.5)		
≥ 51	107 (12.7)	68 (14.5)	21 (11.5)	18 (9.5)		
Marital status					*χ* ^2^ * = *10.350	0.006^∗^
No	167 (19.9)	102 (21.7)	21 (11.5)	44 (23.3)		
Yes	673 (80.1)	367 (78.3)	161 (88.5)	145 (76.7)		
Number of children					*H* = 27.589	< 0.001^∗^
0	111 (13.2)	52 (11.1)	17 (9.3)	42 (22.2)		
1	214 (25.5)	121 (25.8)	43 (23.6)	50 (26.5)		
2	324 (38.6)	180 (38.4)	89 (48.9)	55 (29.1)		
≥ 3	191 (22.7)	116 (24.7)	33 (18.1)	42 (22.2)		
Religion					*χ* ^2^ * = *4.255	0.119
No	693 (82.5)	383 (81.7)	145 (79.7)	165 (87.3)		
Yes	147 (17.5)	86 (18.3)	37 (20.3)	24 (12.7)		
Educational level					*H* = 57.376	< 0.001^∗^
Junior high school and below	287 (34.2)	44 (23.3)	34 (18.7)	209 (44.6)		
Senior high school and junior college	426 (50.7)	120 (63.5)	107 (58.8)	199 (42.4)		
Bachelor's degree and above	124 (15.1)	25 (13.2)	41 (22.5)	61 (13.0)		
Type of health insurance					*H* = 3.607	0.221
New rural cooperative medical system	701 (83.5)	432 (92.1)	113 (62.1)	156 (82.5)		
Medical insurance for urban residents and employees	113 (13.5)	17 (3.6)	69 (37.9)	27 (14.3)		
Out-of-pocket payment	26 (3.1)	20 (4.3)	0 (0.0)	6 (3.2)		
Household per capita monthly income (yuan)					*H* = 173.139	< 0.001^∗^
< 1000	68 (8.1)	20 (4.3)	28 (15.4)	20 (10.6)		
1000–2999	175 (20.8)	55 (11.7)	86 (47.3)	34 (18.0)		
3000–4999	304 (36.2)	234 (49.9)	21 (11.5)	49 (25.9)		
> 5000	293 (34.9)	160 (34.1)	47 (25.8)	86 (45.5)		
Employment status					*χ* ^2^ * = *275.466	< 0.001^∗^
No	566 (67.4)	428 (91.3)	67 (36.8)	71 (37.6)		
Yes	274 (32.6)	41 (8.7)	115 (63.2)	118 (62.4)		
Cancer stage					*H* = 26.809	< 0.001^∗^
0	132 (15.7)	95 (20.3)	17 (9.3)	20 (10.6)		
I	305 (36.3)	176 (37.5)	54 (29.7)	75 (39.7)		
II	403 (48.0)	198 (42.2)	111 (61.0)	94 (49.7)		
Unilateral or bilateral breast disease					*χ* ^2^ * = *36.150	< 0.001^∗^
Unilateral	618 (73.6)	380 (81.0)	127 (69.8)	111 (58.7)		
Bilateral	222 (26.4)	89 (19.0)	55 (30.2)	78 (41.3)		
Type of surgery					*χ* ^2^ * = *9.823	0.007^∗^
Breast-conserving surgery	283 (33.7)	163 (34.8)	45 (24.7)	75 (39.7)		
Mastectomy	557 (66.3)	306 (65.2)	137 (75.3)	114 (60.3)		
Participation competence (score)	—	77.88 ± 8.71	104.45 ± 15.11	112.70 ± 10.47	*H* = 120.486	< 0.001^∗^
Perceived social support (score)	—	36.86 ± 8.68	55.03 ± 11.61	63.91 ± 9.73	*H* = 106.073	< 0.001^∗^
Self-care self-efficacy (score)	—	64.24 ± 12.13	89.59 ± 15.78	96.08 ± 16.51	*H* = 105.350	< 0.001^∗^

^∗^ *p* < 0.01: The difference is statistically significant.

**Table 4:** Variable assignment.

Variable	Assignment method
1. Age (years)	18–30 (0, 0, 1), 31–40 (0, 1, 0), 41–50 (1, 0, 0), ≥ 51 (0, 0, 0)
2. Marital status	No = 0, yes = 1
3. Number of children	0 (0, 0, 1), 1 (0, 1, 0), 2 (1, 0, 0), ≥ 3 (0, 0, 0)
4. Educational level	Junior high school and below (0, 1),senior high school and junior college (1, 0), bachelor's degree and above (0, 0)
5. Household per capita monthly income (yuan)	< 1000 (0, 0, 1), 1000–2999 (0, 1, 0), 3000–4999 (1, 0, 0), ≥ 5000 (0, 0, 0)
6. Employment status	No = 0, yes = 1
7. Cancer stage	0 (0, 1), I (1, 0), II (0, 0)
8. Unilateral or bilateral breast disease	Unilateral = 0, bilateral = 1
9. Type of surgery	Breast-conserving surgery = 0, mastectomy = 1
10. Participation competence	Actual data
11. Perceived social support	Actual data
12. Self-care self-efficacy	Actual data

**Table 5:** Associated factors of breast cancer patients' shared decision-making behavior in surgery (*n* = 840).

Variable	Class 2 vs Class 1				Class 3 vs Class 1				Class 3 vs Class 2			
	*β*	OR	95% CI	*p*	*β*	OR	95% CI	*p*	*β*	OR	95% CI	*p*
(Intercept)	9.399				21.705				12.306			
Age (years)												
18–30	0.747	2.111	0.817∼5.451	0.123	1.095	0.809	0.764∼0.856	< 0.001^∗^	0.348	1.416	0.412∼4.861	0.580
31–40	1.149	3.155	1.192∼8.348	0.021^∗^	1.576	4.834	1.241∼18.831	0.023^∗^	0.427	1.532	0.442∼5.310	0.501
41–50	−0.786	0.456	0.173∼1.199	0.111	1.252	3.496	0.846∼14.543	0.084	2.037	7.669	1.929∼30.488	0.004^∗^
≥ 51	—	—	—	—	—	—	—	—	—	—	—	—
Marital status												
No	−0.946	0.388	0.138∼1.093	0.073	−0.521	0.594	0.114∼3.083	0.535	0.425	1.529	0.325∼7.206	0.591
Yes	—	—	—	—		—	—	—		—	—	—
Number of children	—					—						
0	−1.156	0.315	0.111∼0.894	0.030^∗^	−1.245	0.288	0.054∼1.547	0.147	−0.088	0.915	0.174∼4.826	0.917
1	−0.352	0.704	0.245∼2.018	0.513	0.452	1.572	0.314∼7.880	0.582	0.804	2.234	0.514∼9.707	0.283
2	−0.079	0.924	0.345∼2.472	0.875	0.304	1.356	0.282∼6.518	0.704	0.384	1.468	0.347∼6.026	0.602
≥ 3	—	—	—	—	—	—	—	—	—	—	—	—
Educational level												
Junior high school and below	−0.616	0.540	0.202∼1.444	0.220	4.043	56.992	12.480∼260.571	< 0.001^∗^	4.658	105.470	25.255∼440.463	< 0.001^∗^
Senior high school and junior college	0.019	1.019	0.434∼2.390	0.966	3.835	46.281	11.982∼178.754	< 0.001^∗^	3.816	45.429	13.028∼156.250	< 0.001^∗^
Bachelor's degree and above	—	—	—	—	—	—	—	—	—	—	—	—
Household per capita monthly income (yuan)												
< 1000	2.266	9.644	3.772∼24.661	< 0.001^∗^	2.386	10.867	2.144∼55.080	< 0.001^∗^	0.119	1.127	0.254∼4.993	0.875
1000–2999	2.437	11.437	5.012∼26.100	< 0.001^∗^	0.565	1.759	0.523∼5.912	0.361	−1.872	0.154	0.055∼0.432	< 0.001^∗^
3000–4999	0.449	1.566	0.666∼3.685	0.304	1.367	3.923	1.279∼12.028	0.017^∗^	0.918	2.505	0.904∼6.943	0.078
≥ 5000	—	—	—	—	—	—	—	—	—	—	—	—
Employment status												
No	−1.584	0.205	0.097∼0.432	< 0.001^∗^	−0.063	0.939	0.299∼2.953	0.915	1.521	4.557	1.621∼12.921	0.004^∗^
Yes	—	—	—	—	—		—	—	—	—	—	—
Cancer stage												
0	0.134	1.144	0.368∼3.554	0.816	2.256	15.787	3.197∼75.900	0.016^∗^	2.391	10.720	2.678∼42.907	0.001^∗^
I	−0.659	0.517	0.258∼1.038	0.064	0.140	1.150	0.417∼3.172	0.064	0.799	2.223	0.928∼5.324	0.073
II	—	—	—	—	—	—	—	—	—	—	—	—
Unilateral or bilateral breast disease												
Unilateral	0.506	1.659	0.895∼3.076	0.108	0.170	1.185	0.412∼3.409	0.752	−0.336	0.714	0.266∼1.922	0.506
Bilateral	—	—	—	—	—		—	—	—	—	—	—
Type of surgery												
Breast-conserving surgery	−1.068	0.344	0.172∼0.688	0.003^∗^	0.517	1.676	0.595∼4.722	0.328	1.584	4.876	1.824∼13.033	0.002^∗^
Mastectomy	—	—	—	—	—		—	—	—	—	—	—
Participation competence	−0.083	0.921	0.886∼0.957	< 0.001^∗^	−0.212	0.809	0.764∼0.856	< 0.001^∗^	−0.10	0.878	0.837∼0.922	< 0.001^∗^
Perceived social support	0.065	1.068	1.036∼1.100	< 0.001^∗^	0.024	1.024	0.987∼1.062	0.206	−0.042	0.959	0.933∼0.987	0.004^∗^
Self-care self-efficacy	−0.113	0.893	0.857∼0.931	< 0.001^∗^	−0.167	0.846	0.894∼0.901	< 0.001^∗^	−0.054	0.948	0.908∼0.990	0.015^∗^

^∗^ *p* < 0.05: The difference is statistically significant.

## Data Availability

The data that support the findings of this study are available from the corresponding authors upon reasonable request.
