# Correction to “Shared Decision‐Making Behavior in Surgery Among Early Breast Cancer Patients and Associated Factors Using COM‐B Model: A Latent Profile Analysis”

**DOI:** 10.1155/jonm/9762416

**Published:** 2026-05-29

W. Zhang, Y. Weng, X. Zhang, et al., “Shared Decision‐Making Behavior in Surgery Among Early Breast Cancer Patients and Associated Factors Using COM‐B Model: A Latent Profile Analysis,” *Journal of Nursing Management*, 2025, 2347796, https://doi.org/10.1155/jonm/2347796.

In the article, there are errors in Table 1 and the discussion section. In Table 1, the values stated for the LMR, BLRT and Entropy columns are incorrect. The correct Table 1 is shown below:

**TABLE 1:** Fitting indices of the latent profile model for shared decision‐making behaviour in surgery.

Model	*k*	AIC	BIC	aBIC	*p*		Entropy	Class probability
					LMR	BLRT		
1	24	21,615.538	21,729.140	21,652.924				
2	37	17,152.490	17,327.626	17,210.126	< 0.001	< 0.001	0.969	0.402/0.598
3	50	16,559.920	16,796.591	16,637.807	0.020	< 0.001	0.980	0.217/0.557/0.226
4	63	15,967.403	16,265.607	16,065.540	0.082	< 0.001	0.992	0.134/0.117/0.225/0.524
5	76	15,035.028	15,394.766	15,153.415	0.585	< 0.001	0.991	0.280/0.109/0.112/0.206/0.293

*Note:* aBIC, sample size–adjusted Bayesian information criterion; LMR, Lo–Mendell–Rubin adjusted likelihood ratio test.

Abbreviations: AIC, Akaike information criterion; BIC, Bayesian information criterion; BLRT, bootstrap likelihood ratio test.

^∗^ *p* < 0.05: The n‐category model is significantly superior to the n‐1‐category model.

Lastly, the fourth paragraph of the discussion section should be corrected from:

“In addition, among the factors identified based on the COM‐B model, the results indicated that participation competence, perceived social support, and self‐care self‐efficacy were significant protective factors for SDM behavior in surgery across two different subtypes, with the exception of self‐care self‐efficacy between Class 3 and Class 1 (*p* > 0.05). Self‐care self‐efficacy also functioned as a risk factor between Class 2 and Class 1. Patients with high self‐care self‐efficacy tend to consider not only their medical conditions but also various factors such as economic and social implications, leading to increased hesitation and complexity when making decisions. Higher self‐care self‐efficacy is associated with greater self‐confidence, which may encourage patients to question and evaluate the decisions made by health professionals, potentially impacting their engagement in treatment SDM behavior [28].”

To:

“In addition, among the factors identified based on the COM‐B model, the results indicated that participation competence, perceived social support, and self‐care self‐efficacy were significant protective factors for SDM behavior in surgery across two different subtypes, with the exception of perceived social support between Class 3 and Class 1 (*p* > 0.05). Perceived social support also functioned as a risk factor between Class 2 and Class 1. Patients with high perceived social support tend to consider not only their medical conditions but also various factors such as family and social implications, leading to increased hesitation and complexity when making decisions. Higher perceived social support is associated with greater family dependence, which may prompt patients to question and evaluate the decisions made by health professionals, potentially impacting their engagement in treatment SDM behavior [28].”

We apologize for these errors.

